# Cyclic Evolution of Coronal Fields from a Coupled Dynamo Potential-Field Source-Surface Model

**DOI:** 10.1007/s11207-015-0831-8

**Published:** 2015-12-29

**Authors:** Mausumi Dikpati, Akshaya Suresh, Joan Burkepile

**Affiliations:** High Altitude Observatory, National Center for Atmospheric Research, 3080 Center Green, Boulder, CO 80307-3000 United States; Department of Astronomy, Yale University, New Haven, CT United States

**Keywords:** Corona, structure, Corona, model, Solar cycle, Velocity fields, interior

## Abstract

The structure of the Sun’s corona varies with the solar-cycle phase, from a near spherical symmetry at solar maximum to an axial dipole at solar minimum. It is widely accepted that the large-scale coronal structure is governed by magnetic fields that are most likely generated by dynamo action in the solar interior. In order to understand the variation in coronal structure, we couple a potential-field source-surface model with a cyclic dynamo model. In this coupled model, the magnetic field inside the convection zone is governed by the dynamo equation; these dynamo-generated fields are extended from the photosphere to the corona using a potential-field source-surface model. Assuming axisymmetry, we take linear combinations of associated Legendre polynomials that match the more complex coronal structures. Choosing images of the global corona from the Mauna Loa Solar Observatory at each Carrington rotation over half a cycle (1986 – 1991), we compute the coefficients of the associated Legendre polynomials up to degree eight and compare with observations. We show that at minimum the dipole term dominates, but it fades as the cycle progresses; higher-order multipolar terms begin to dominate. The amplitudes of these terms are not exactly the same for the two limbs, indicating that there is a longitude dependence. While both the 1986 and the 1996 minimum coronas were dipolar, the minimum in 2008 was unusual, since there was a substantial departure from a dipole. We investigate the physical cause of this departure by including a North–South asymmetry in the surface source of the magnetic fields in our flux-transport dynamo model, and find that this asymmetry could be one of the reasons for departure from the dipole in the 2008 minimum.

## Introduction

**Figure 1:**
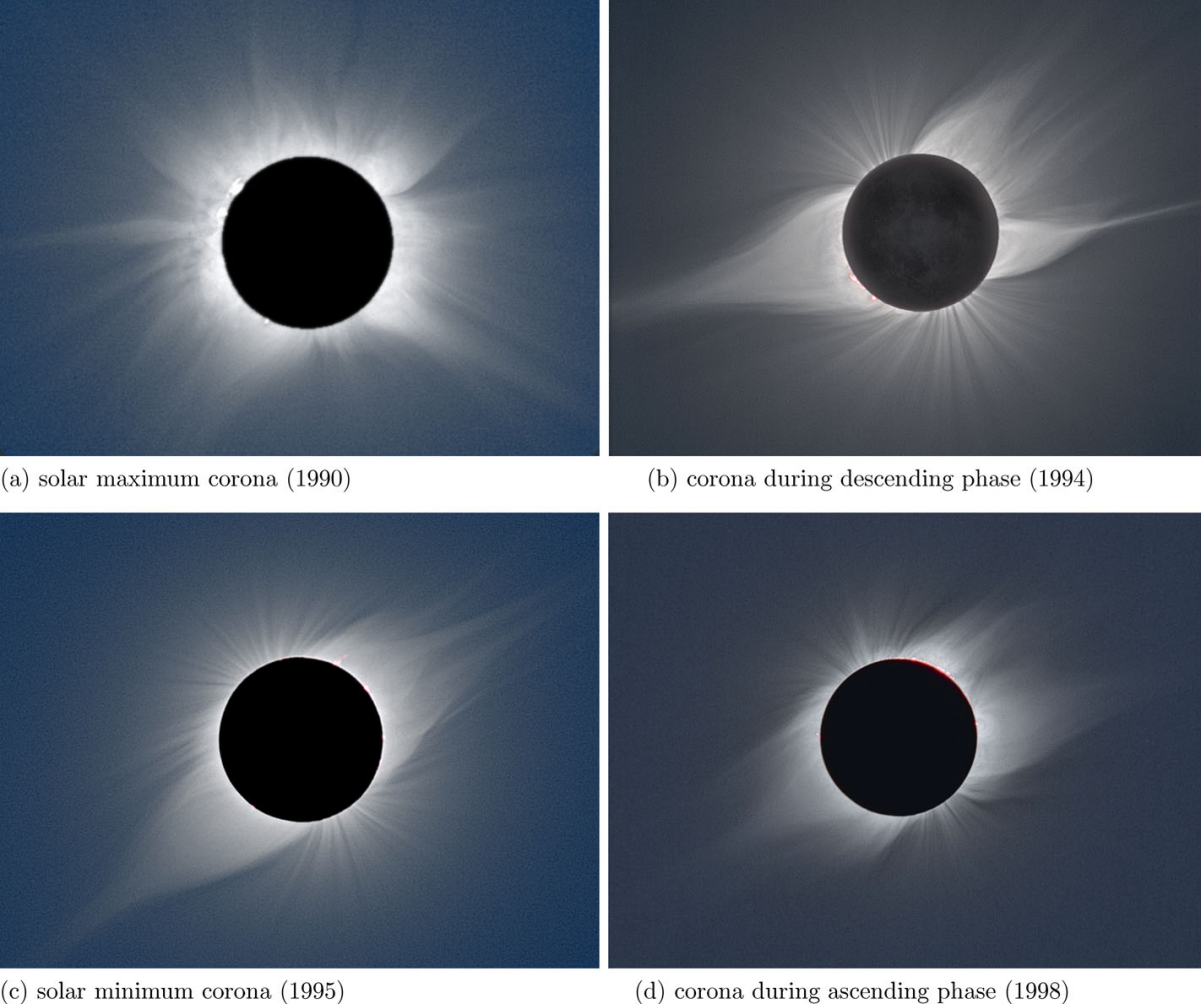
Images of total solar-eclipse corona at four different phases of a solar cycle. (a) Corona during Cycle 22 maximum, (b) corona during the declining phase of Cycle 22, (c) corona at solar minimum at the end of Cycle 22, (d) corona at the ascending phase of Cycle 23. Source of all images is the MMV project website of Prof. M. Druckmüller (http://www.zam.fme.vutbr.cz/~druck/eclipse/).

In most solar cycles, the coronal structure seen in white-light coronagraphs and solar-eclipse images evolves through a sequence of patterns in which the relative strength of the axial dipole and higher multipoles varies with cycle phase. It is well known that white-light structures in the corona reveal its global magnetic structure, including where field lines are open (sources of the solar wind) and closed (locations of ‘streamers’ containing closed magnetic arches). Figure 1 shows a typical example, constructed by including various total solar-eclipse pictures which are processed by Prof. M. Druckmüller using the software developed (Morgan and Druckmüller 2014) for the project of Mathematical Methods of Visualization of Solar Corona (MMV). Figure 1 shows images of the Sun during total solar eclipses in four different phases of Solar Cycle 22. Near the maximum in 1990, we can see that the corona, as revealed by the total solar eclipse on 22 July 1990, picture taken in Russia, looks almost spherically symmetric (panel a). By 1994 the cycle is in its descending phase (panel b). In this image, taken in Bolivia during the total solar eclipse on 3 November 1994, we start seeing the appearance of dipolar structure. The year 1995 was near solar minimum. In panel c, produced from the total solar-eclipse image during 24 October 1995, taken in India, the dipole structure is extremely clear. By 1998 (panel d), we are in the ascending phase of Cycle 23 and we can see, from the image during the total solar eclipse on 26 February 1998, taken in Aruba, the evolution of the strong dipole structure into a smoother, more spherically symmetric pattern once more.

While the evolution of the coronal structure with cycle phase described above is fairly typical, it is not always the case. For example, the minimum corona between Cycles 23 and 24 was decidedly different from a pure dipole. There have been a number of observational studies that relate global patterns of solar magnetic fields to spherical harmonics and their extensions into the corona as determined using potential-field models without or with a poloidal source surface where the field lines open up into the interplanetary medium (Wang, Lean, and Sheeley 2000; Knaack and Stenflo 2005; Robbrecht *et al.*^2010^; Robbrecht and Wang 2012; Petrie 2013; Petrie and Haislmaier 2013).

It is generally agreed that the origin of coronal magnetic structures is the magnetohydrodynamic dynamo that operates in the solar convection zone and tachocline (see, *e.g.*, Pinto *et al.*^2011^). Magnetic fields generated and amplified there reach the photosphere, and imprint their patterns on the corona above. Therefore, if we are to understand the origin of the sequence of coronal structures observed over a typical solar cycle, together with the variations from cycle to cycle, we must link the solar dynamo to the corona in a quantitative way. This is a formidable task. As of now no fully 3D global MHD model of the solar dynamo that has been calibrated for the Sun yields plausible ‘solar-cycle’ type behavior. But there are other, somewhat less comprehensive MHD models that have proved extremely valuable for simulating solar dynamo properties and which create magnetic patterns that are used to create solar-cycle-like sequences of coronal structures. These models are of basically two types: surface transport models (Wang, Sheeley, and Lean, 2000, 2002; Wang, Lean, and Sheeley 2002; Wang and Sheeley 2003; Baumann *et al.*^2004^; Wang, Robbrecht, and Sheeley 2009; Wang *et al.*^2010^) and axisymmetric flux-transport dynamo models (Pinto *et al.*^2011^; DeRosa, Brun, and Hoeksema 2012; Dikpati 2013).

In this article we will use a Sun-calibrated axisymmetric flux-transport dynamo to address the question of how the solar dynamo generates the global coronal patterns that are observed. Since the corona has 3D structures, and the surface flux-transport models referenced above and our dynamo model are axisymmetric, they are not able to explain all aspects of the coronal structure through a cycle. But they can give insight about some of the most prominent parts of these structures, namely the axial dipole and higher-order multipoles. Patterns that no axisymmetric model can simulate directly are what are sometimes referred to as the equatorial dipole and quadrupole. These correspond, respectively, to magnetic patterns that have longitudinal wavenumbers $m=1,2$. A non-axisymmetric surface-transport or flux-transport dynamo model would be needed for these.

Given that the solar dynamo is fully 3D, nonlinear, and turbulent, there are inherent limitations to the essentially linear concept of ‘dynamo modes’ identified with any particular spherical harmonic or other function. On the other hand, any magnetic pattern on the surface of a sphere can be represented by a series of such spherical harmonics (see, *e.g.*, Knaack and Stenflo 2005; DeRosa, Brun, and Hoeksema 2012) and these can be related to coronal observations (Petrie 2013). Furthermore, it is well known that the higher-order harmonics fall off in amplitude faster with radius (see Equation (4)), so it is inevitable that if we look far enough above the surface, the lower order harmonics, or global ‘dipoles’, dominate in the series representation. Therefore, even if they are not true ‘modes’ in the linear sense, it still makes sense to develop theoretical explanations for their amplitude and orientation.

An inherent property of spherical harmonic representations of data on a sphere is that they are divided into two classes of harmonics, one symmetric and the other antisymmetric about the Equator. The axial dipole of the Sun is identified with the antisymmetric class, since the North and South polar fields are generally opposite in sign. However, they are rarely equal in amplitude, and they generally do not reverse at the same time. This lack of precise antisymmetry between North and South implies the axisymmetric field of the Sun must include at least a quadrupolar as well as dipolar component, since quadrupoles are symmetric about the equator, so they have the same magnetic field sign as well as amplitude at the North and South poles. It follows that even coronal structures averaged over longitude around the Sun will still show differences between the North and South hemispheres. Therefore any dynamo or surface flux-transport model used to create coronal structures will need to be able to generate both dipolar- and quadrupolar-type fields at the same time. At certain cycle phases, still higher multipoles of either or both symmetries may also be needed.

Since it is well established that in most of the observed solar cycles there are significant differences between the magnetic activity in the North and South, as well as in the timing of maxima and minima there (see, *e.g.*, Dikpati *et al.*^2007^). It is plausible to assume that the magnetic coupling between North and South is relatively weak. Nevertheless there is enough coupling that, at least for the past few centuries for which observations are available, the two hemispheres do not get out of phase so much that one hemisphere is going through minimum while the other is near maximum. This reasoning reinforces the notion that in all models both symmetries about the equator need to be included.

Given the reasoning above, it is particularly important to consider possible sources for differences in dynamo action between the North and South hemispheres that could lead to corresponding differences in coronal structure. Two obvious possibilities are differences between North and South in differential rotation and meridional circulation, but also differences in the emergence and twisting of the magnetic flux generated within the convection zone. Of these, differences in differential rotation seem the least important, since they are so small (torsional oscillations have amplitude no more than a few tenths of a percent of the solar rotation), but meridional circulation is observed to vary by perhaps a factor of two, and can have different profiles in the North and South hemispheres (Haber *et al.*^2002^; Ulrich 2010; Komm *et al.*^2013^). Magnetic flux emergence can also differ between hemispheres over several years by a factor of two. Belucz, Forgács-Dajka, and Dikpati (2013) and Belucz and Dikpati (2013) have analyzed the effects of such differences on butterfly diagrams and polar fields in solutions to the flux-transport dynamo model. We have begun studying the coronal effects of both these differences with the same model.

## Methodology

We adopt a two part strategy for relating observed coronal structures to magnetic fields generated by a flux-transport dynamo in the domain above the photosphere. Both parts of the strategy involve the use of associated Legendre polynomials $P^{l}_{n}$. Why these polynomials? Because they are the natural solutions for the vector potential for the large-scale poloidal magnetic fields in the global-scale inner corona where the magnetic fields are large enough that the fields cannot be very far from being a potential field, because if they were the electromagnetic body force would be much larger than all other forces and be unbalanced. The coronal material that is there is frozen-in to the magnetic field lines, so that white-light coronal brightness reveals the field patterns. This is usually the case in the corona below about 2.5 solar radii from the solar center, above which the solar wind becomes strong enough to stretch out the poloidal field and ‘open’ it into interplanetary space. The relatively static, slowly changing fields are determined largely by the lower boundary condition of large-scale photospheric fields, whether observed or generated by a dynamo model, and by the fields turning radial at the outer edge of the domain due to the solar wind. An exception to these conditions occurs when, for example, a coronal mass ejection is triggered, but consideration of such transient events in modeling the large-scale coronal structure is beyond the scope of the present paper. Placing the source-surface at 2.5 solar radii is a common choice, although there are times in some solar cycles during which a larger radius would be appropriate (Zhao and Hoeksema 2010), or a smaller radius (Lee *et al.*^2011^). It has also been demonstrated that source surface models can often do as well as full 3D MHD models in simulating coronal structures (Riley *et al.*^2006^).

It is well known that white-light structures in the corona tend to be frozen into the magnetic field due to the high conductivity in the corona and therefore, are tracers of magnetic field lines under most conditions. Coronal matter is constrained to be ‘tied’ to the poloidal field. These field lines essentially become density contours. We take advantage of this property to qualitatively ‘fit’ observed white-light images of the corona to short series of polynomials with the weights chosen to maximize the correspondence with the observations. This fit is very simply done, namely by eye estimation, *i.e.*, by adjusting the relative amplitudes of the first several Legendre polynomials in a series solution for the poloidal vector potential [$A$] until the contours of [$A$] most closely resemble the observed white-light coronal brightness pattern chosen. Then we compare the results from this exercise carried out for various phases of the past three solar cycles with coronal fields generated by a Sun-calibrated flux-transport dynamo model that is coupled to a potential-field source-surface (PFSS) model (see Hoeksema and Scherrer 1986; Wang and Sheeley 1992; and references cited therein).

The fitting to observations clearly has limitations imposed by the assumption of axisymmetry or independence in longitude. While coronal structures may have considerable longitudinal extent, they are not generally axisymmetric, so the relative weights of the Legendre polynomials could be somewhat different depending on the precise location of the coronal structure relative to the plane of the disk. Intuitively, we reason that a coronal feature either slightly in front of or behind the disk plane will tend to favor slightly higher amplitudes for higher polynomial index, since they decline more rapidly with radius (see Equation (4) below).

In quantitative mathematical terms, in both the convection zone and the corona above, the magnetic field is given by the MHD induction equation:

1 $$ \frac{\mathbf{\partial B}}{\partial t}= {\nabla}\times(\mathbf{V}\times\mathbf{B} -\eta{ \nabla}\times\mathbf{B}), $$

in which $\mathbf{B}$ is the magnetic field, $\mathbf{V}$ the velocity and $\eta$ is the magnetic diffusivity.

The components of $\mathbf{B}$ in the meridional plane, or the poloidal field that coronal structures outline, can be represented as the curl of a vector potential [$A$], if we assume an axisymmetric model, so there is no ${\phi}$ dependence to the field and vector potential components. In that case, the magnetic field can be written as the vector sum of the toroidal and poloidal components as:

2 $$ \mathbf{B}(r,\theta,t)=B_{\phi}(r,\theta,t)\hat{{e}}_{\phi}+{\nabla}\times \bigl[A(r,\theta,t) \hat{{e}}_{\phi}\bigr]. $$

Because of the fact that the corona is almost completely ionized, the mean free path of coronal particles is determined mainly by the interaction of ionized particles with the ambient magnetic field. The mean free path along field lines is large compared to that perpendicular to the fields, but both are short compared to the typical scale of magnetic and density structures in the corona, so the MHD equations apply well, even though the particle density is very low (Wiegelmann, Petrie, and Riley 2015). The magnetic diffusivity of the corona is also determined by the mean free path; it is large enough inside of closed coronal structures, while persistent large-scale flows there are small enough that the diffusive term in Equation (1) is much larger than the inductive term. Except during a reconnection event, which we are not considering here, the time rate of change of the field in Equation (1) is also small.

Further out in the corona, where the solar wind is strong enough to open many of the field lines, the induction term in Equation (1) is no longer small, but this effect is captured in a simple way by adding the source surface where by assumption the field lines become radial. This source surface therefore contains electric currents in the surface that by Ampere’s law match with the radial fields. But everywhere inside this surface the poloidal field is still a potential field, and nonpotential outside. We have considered this type of structure in our dynamo simulations. But for practical reasons, when matching the coronal observations to contours of the poloidal potential we omit the source surface. Including it would add to the degrees of freedom to constrain in the fit, and also it is hard to tell from white-light observations where it should be, since in open structures the coronal density is very low. Here we choose to avoid such complications.

Under these conditions we can calculate the vector potential [$A$] in the corona from the diffusion term in Equation (1) alone. In this case the magnetic field is a potential field determined by a form of Laplace’s equation; there are no currents anywhere in the domain:

3 $$ \biggl(\nabla^{2}-\frac{1}{r^{2} \sin^{2}\theta} \biggr)A=0. $$

In the current-free approximation, the solution for this equation can be written as:

4 $$\begin{aligned} &A(r\geq R_{\odot},\theta,t)=\sum_{n} \frac{a_{n}(t)}{r^{n+1}}P_{n}^{l}(\cos \theta), \end{aligned}$$

5 $$\begin{aligned} &a_{n}(t)=\frac{(2n+1)R_{\odot}^{n+1}}{n(n+1)} \int_{0}^{\pi /2}A(r=R_{\odot},\theta,t) P_{n}^{l}(\cos\theta)\sin\theta \,\mathrm{d}\theta, \end{aligned}$$

where $P_{n}^{l}(\cos\theta)$ is the associated Legendre polynomial. The derivative of [$A$] at the solar surface

6 $$ \frac{\partial{A}}{\partial{r}}\bigg\vert _{(r=R_{\odot})}= -\sum _{n}\frac{(n+1)a_{n}(t)}{R_{\odot}^{n+2}}P_{n}^{l}(\cos \theta). $$

The index $l$ is usually associated with non-axisymmetric harmonics with longitudinal wave number $l$, but here it is $l=1$ due to the $1/(r^{2} \sin^{2} \theta)$ term in Laplace’s equation. The number of nodes in the magnetic pattern in latitude between the poles is given by $(l-n)$. As a first step in our methodology, we choose the coefficients of the associated Legendre polynomials to create solutions that match observations above the photosphere without considering the conditions at the photosphere.

The second step in our strategy is to implement a dynamo–PFSS model in which we no longer use the current-free approximation; instead we add the influence of the solar wind from a certain radius, $r=R_{\mathrm{ss}}$. Thus the large-scale poloidal field is potential from $r=R$ to $r=R_{\mathrm{ss}}$, and beyond that it is radial due to the stretching by the solar wind. Therefore, instead of using a simple potential field, we represent, following Altschuler and Newkirk (1969), the dynamo-generated poloidal-field vector potential [$A$] with the following form of [$A$] above the photosphere:

7 $$ A(R \le r \le R_{\mathrm{ss}}, \theta, t)= \Sigma_{n} {a_{n}(t) \over r^{n+1}} {P_{n}}^{1} (\cos\theta) \biggl[c_{n} + (1-c_{n}) { \biggl(\frac{r}{R} \biggr)}^{2n+1} \biggr]. $$

The $c_{n}$ coefficients are found by requiring $B_{\theta}$ to vanish at $r=R_{\mathrm{ss}}$, which gives

8 $$ \frac{1}{c_{n}} = 1 + \frac{n}{n+1} { \biggl(\frac{R}{R_{\mathrm{ss}}} \biggr)}^{2n+1}. $$

Therefore, the boundary condition at the surface becomes

9 $$ \frac{\partial{A}}{\partial{r}}\bigg\vert _{(r=R_{\odot})}= -\sum _{n}\frac{(n+1)a_{n}(t)}{R_{\odot}^{n+2}}P_{n}^{l}(\cos \theta) \biggl[c_{n} - { \biggl(\frac{n}{n+1} \biggr)}(1-c_{n}) \biggr]. $$

## Results

**Figure 2:**
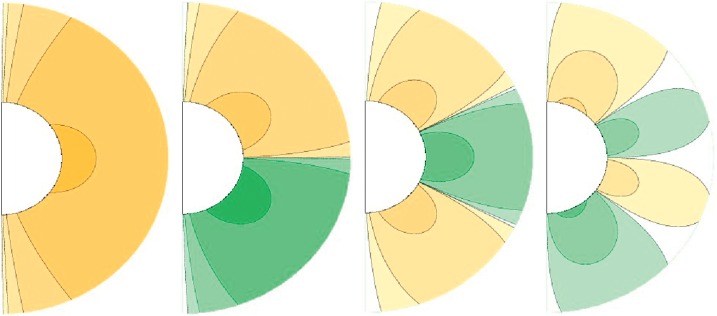
Associated Legendre polynomials of the first order. From left to right images are first degree (${n=l=1}$), second degree ($n=2$, $l=1$), third degree ($n=3$, $l=1$), and fourth degree ($n=4$, $l=1$).

To plot the calculated field lines, we start with the components of the poloidal magnetic field in spherical polar coordinates. We compute field lines by calculating $B_{r}$ and $B_{\theta}$ from Equation (2). Equivalently, we can plot contours of the quantity $Ar\sin{\theta}$. Figure 2 shows representations of the first four associated Legendre polynomials of the first order ($l=1$) and $n=1,2,3,4$, respectively.

The orange color represents positive potential (clockwise field arrows) and green represents negative potential (counter-clockwise field arrows). We see that $n=1$ gives a dipolar structure, $n=2$ gives a quadrupolar structure, $n=3$ a hexapolar, and $n=4$ an octopolar form. $n=1,3$ are antisymmetric about the Equator in field direction and $n=2,4$ symmetric in field direction.

Taking linear combinations of these basic structures yields more complicated-looking solutions. The goal is to find the recipes for the coefficients $a_{n}^{1}$ that add the right amount of the different types of multipoles to match the white-light coronal observations. Since we cannot tell from observations whether a structure is positive or negative, we choose coefficients only to match the intensity of the white-light structures.

**Figure 3:**
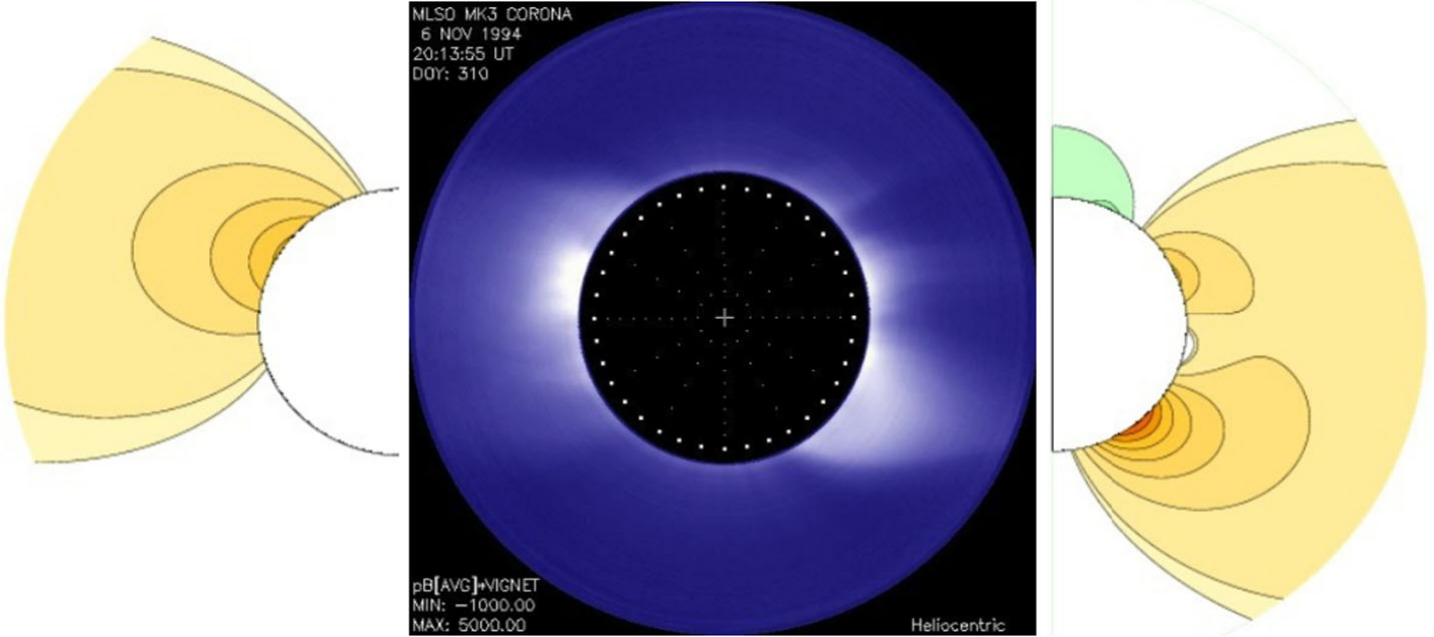
A typical solar corona, derived from MLSO image during November 1994, is placed at the center. On its left and right are theoretically constructed structures using Legendre polynomial decompositions.

We illustrate our method by showing in Figure 3 a white-light coronal image from Mauna Loa Solar Observatory (MLSO) for November 1994, flanked on the left and right by potential-field structures containing combinations of multipoles that produce structures that most closely resemble the observations. In this example, which is late in Cycle 22, the East and West limb patterns are somewhat different, indicating some longitudinal dependence. But the differences are not large, and we are able to choose coefficients that favor the dipole term with small amounts of some higher multipoles. The coefficients from each limb do almost match, which is what we would expect.

**Figure 4:**
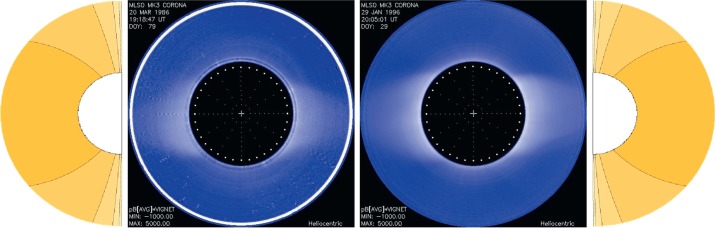
Two typical solar minimum coronae during 1986 and 1996 (two middle frames). Leftmost and rightmost frames are given by ${A=\frac{3P_{1}^{1}}{r^{2}}}$ showing a largely axisymmetric corona.

Figure 4 shows polynomial fits to the minimum coronae in 1986 and 1996. We see that we can get good fits using the same dipole amplitude for both limbs for both minima. The ${P_{1}^{1}}$ term dominates at minimum.

**Figure 5:**
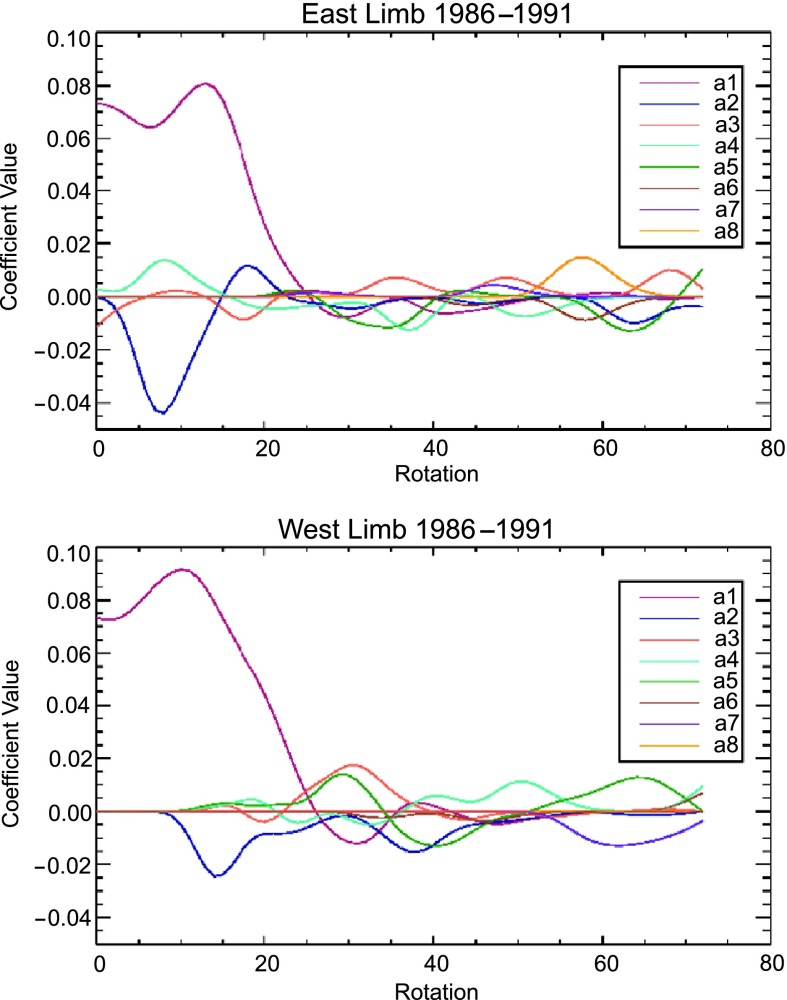
Time evolution of different associated Legendre polynomial coefficients over time, separately in the East (top panel) and West (bottom panel), for the years 1986 – 1991 at the source-surface height of $2.5 R_{\odot}$.

To show how the relative amplitudes of the associated Legendre polynomials evolve over one cycle from minimum to maximum, we chose the time span between 1986 and 1991. Figure 5 shows the time changes in the relative amplitudes that give best fits to MLSO images for each rotation between 1986 and 1991. We see clearly the rapid decrease in the relative amplitude of the dipole moment ($a_{1}$ coefficients) with the rise in solar activity beginning in 1987, for both the East (upper panel) and West (lower panel) limbs. This happened due to the rapid rise in new cycle flux emergence at sunspot latitudes, which weakens the dipole moment (Wang, Sheeley, and Rich 2000). The ${a_{1}}$ coefficient dominates for the first few rotations and then rather rapidly approaches zero while the other coefficients fluctuate.

The results near minimum are similar to those obtained in previous studies (see, *e.g.*, Petrie 2013). It is interesting to note that during the ascending phase and near solar maximum, no multipole appears to dominate. Rather, they all appear to fluctuate almost sinusoidally, with approximately similar amplitudes, though different periods. In Figure 5 we have smoothed the trend lines (a running three-rotation average with equal weights) and taken the values at the source-surface height, $r=2.5 R_{\odot}$, to show the relative influence of each coefficient at a particular location. Absolute values for the coefficients cannot be determined here, as we are not using magnetogram data. Further, there is a clear difference between the East and West limbs, indicating, as we have discussed above, the presence of longitude-dependent coronal structures. We do see that the variation is greatest at solar minimum and least at solar maximum.

**Figure 6:**
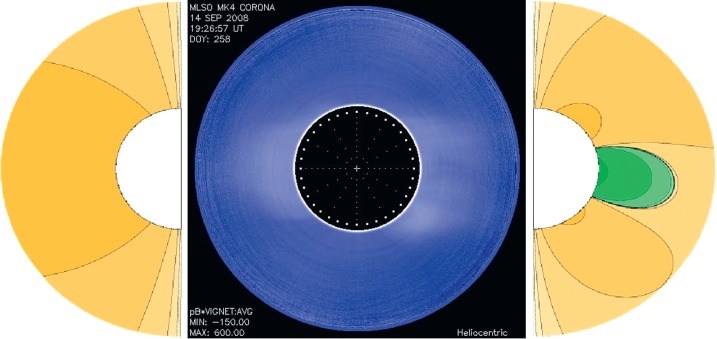
The solar minimum in 2008: The East limb is given by ${A=\frac{2P_{1}^{1}}{r^{2}}}$ and West limb is given by ${A=\frac{0.3P_{1}^{1}}{r^{2}}+\frac{1.7P_{2}^{1}}{r^{3}}+\frac{35P_{3}^{1}}{r^{4}}}$.

**Figure 7:**
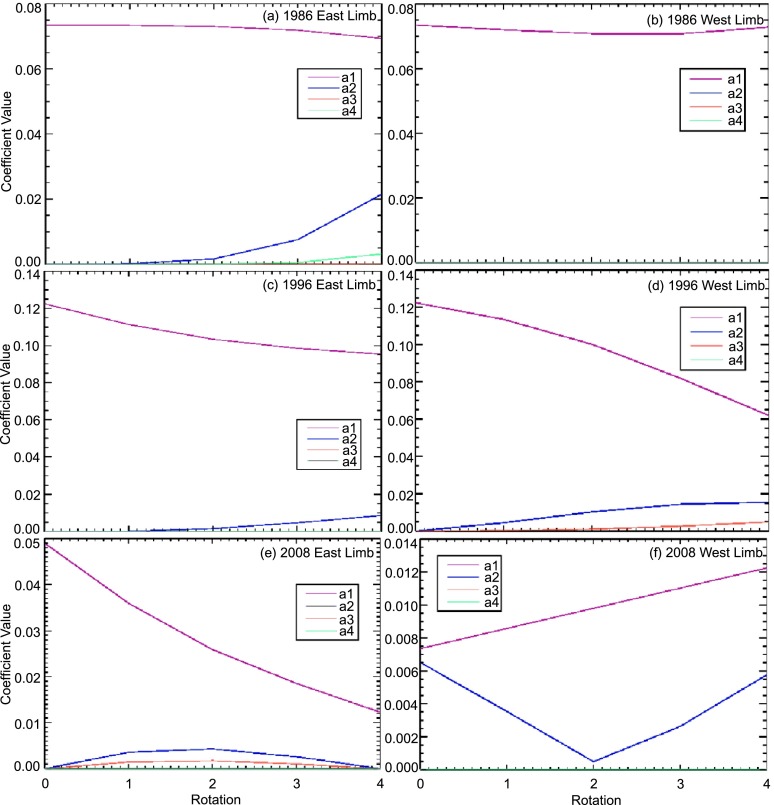
East and West limb coefficients during three solar minima (1986, 1996, 2008). In all three years, we see that the ${a_{1}}$ coefficients are dominant, though they are less prominent in 2008. 2008 is the only time when higher-order coefficients are significant, though even then no coefficients of order greater than four were necessary to fit the observations.

Next we compare the solar minima of three different solar cycles: 21, 22, and 23. To do this, we matched models to images from 1986 and 1996 (Figure 4) and 2008 (Figure 6) from MLSO, and plot the polynomial relative amplitudes for East and West limbs for five consecutive rotations at each minimum in Figure 7. We have again smoothed the trend lines and taken the source-surface height $r=2.5 R_{\odot}$ to show the relative influence of each coefficient at the same distance from the Sun. When looking at solar minima, we expect a predominantly dipolar structure with small amounts of multipoles that increase as we enter the ascending phase. A sustained dipole structure was very clearly seen for a sample of five consecutive rotations from 1986 to 1996, with high ${a_{1}}$ coefficients for both East and West limbs and almost all coefficients close to zero for the multipoles. The one exception is in the ${a_{2}}$ coefficient in the West limb in 1996. These are nonzero values because here the dipole was tilted toward the poles, rather than coming out straight from the Equator. However, the ${P_{2}^{1}}$ term is still not the dominant term here. After these five rotations, multipoles start to emerge slightly both in 1986 and 1996. This change occurs as the Sun exits the minimum and enters the ascending phase. Thus, these results are exactly what one would expect. The disappearance of dipole dominance is rather fast, most of it occurring in between five and ten rotations.

The year 2008 saw a very different solar minimum. In Cycle 23, there was never a rotation in which both limbs were dipoles. Thus, we see that the multipoles are present and significant throughout. The $a_{2}$ coefficient is significant, particularly in the West limb. The difference in $a_{2}$ amplitudes in the East and West limbs again indicates longitude-dependent coronal structures, but there is an average longitude-independent departure from a simple dipole at this time. We also see that the amplitude of this coefficient varies significantly from one rotation to the next even during a solar minimum, showing that this minimum is more dynamic than the previous ones. However, this set is still clearly a solar minimum, since higher-order multipoles, *i.e.*, above ${P_{4}^{1}}$, were not needed to match the observations.

In the next section we use simulations from a calibrated solar flux-transport dynamo model to give a possible explanation for the difference between the most recent minimum and the two that preceded it.

## Coronal Structure from a Dynamo Model

The flux-transport dynamo model ingredients and the parameters we have used are the same as described in Dikpati *et al.* (2010) and Dikpati (2011). Instead of applying a potential-field boundary condition at the top of the convection zone, we have instead solved the equations for fields in the corona by requiring these fields to become radial at a radius of $2.5 R_{\odot}$. Thus we have coupled our flux-transport dynamo model to a potential-field source-surface (PFSS) model. While this change in upper boundary condition adds greater realism to the coronal field lines, and therefore, in principle, a better comparison with observations, we judge that it will change the patterns associated with the lowest Legendre polynomials the most. We have not analyzed the differences in the field lines from the two forms of the dynamo in detail, but we do not expect them to be significant for all patterns beyond the dipole. The dynamo itself freely produces field patterns on the top of the dynamo domain at the photosphere, governed by the interior ingredients, and those field patterns at the upper boundary of the dynamo model become the imposed starting lower boundary fields of the PFSS model of the corona. This method also has limitations, in that there is no reason to believe the source surface should be at the same radius for all latitudes and longitudes.

We have done multiple simulations of the solar dynamo including magnetic structures in the corona, by fitting associated Legendre polynomials to the surface poloidal fields of the dynamo, and then calculating the field lines in the corona in the same way as used to fit the white-light coronal observations. Unlike in the observational case, the field lines on the East and West limbs are mirror reflections of each other, so we need only to show one hemisphere; we choose the East.

We represent the Babcock–Leighton surface poloidal source by a specified function of latitude in each hemisphere, one whose shape is typical of the ‘shape’ of a sunspot cycle (faster and shorter ascending phase, slower and longer descending phase) seen in sunspot number or area plots. We do two simulations, one for which the surface source is antisymmetric about the equator, and another that includes an asymmetry between hemispheres. For a fixed surface source, we take a profile that declines from its peak to zero at a higher latitude in the North than in the South, capturing the fact that in Cycle 23 the sunspots ceased to be seen in the North about two years before they disappeared in the South. The higher latitude (ascending phase and maximum phase) part of the cycle remains antisymmetric about the Equator.

**Figure 8:**
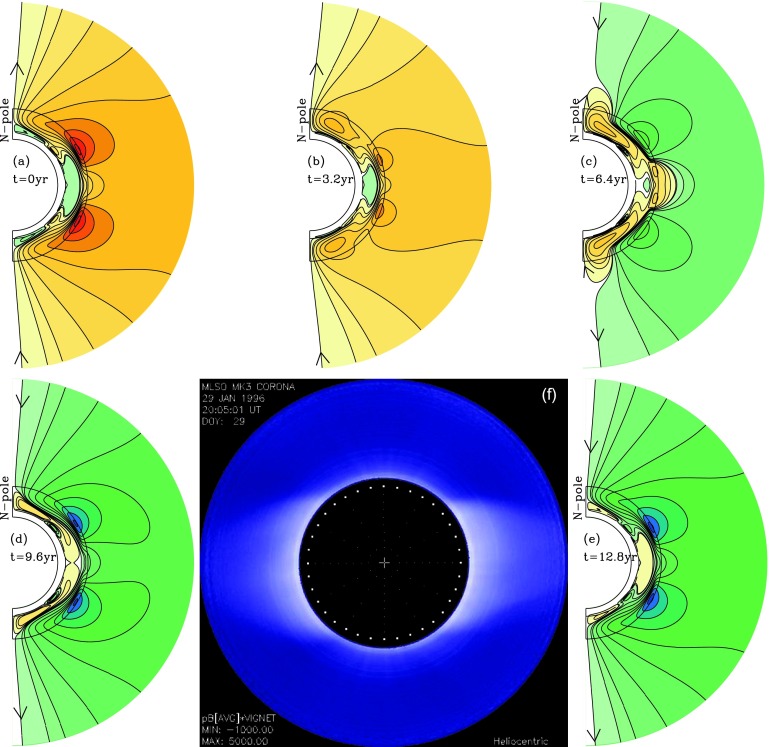
Reference case sequence of dynamo-generated coronal field lines at 3.2 year intervals of a simulated solar cycle (panels a – d, f), compared with the white-light corona of January 1996, late descending phase (panel e). (a) is for the descending phase, (b) the minimum, (c) the ascending phase and (d) the maximum.

**Figure 9:**
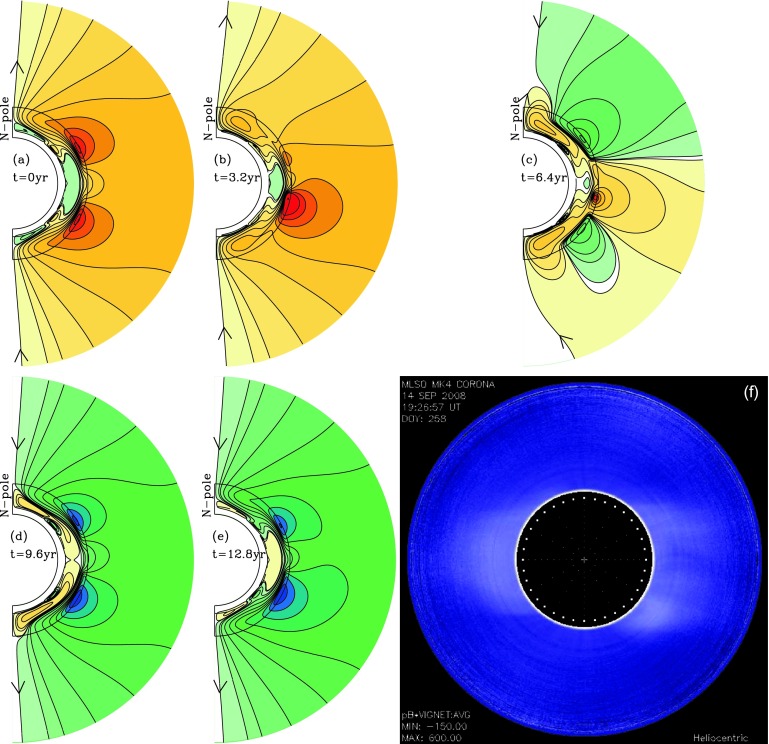
North–South asymmetry-case sequence of dynamo-generated coronal field lines at 3.2 year intervals of a simulated solar cycle (panels a – e; (a) for descending phase, (b) for minimum, (c) for ascending phase, (d) for maximum, (e) for late descending phase and minimum), compared with the white-light corona of September 2008, the atypical minimum corona (panel f).

The simulation starts during the descending phase of the cycle in which the asymmetry between hemispheres is introduced and is continued for one sunspot cycle, 12.8 years in this case. Figures 8 and 9 show our results. All five frames show the resulting poloidal-field lines, sampled at intervals of 3.2 years, for both the convection zone and the corona out to the source surface. Since our focus is on what coronal field lines, *i.e.*, the large-scale poloidal fields, are produced as a function of cycle phase, we omit the corresponding plots of toroidal-field amplitude contours for the convection zone.

The time spacing of the images is such that in each case, the first image is for the descending phase, the second for the minimum, the third for the ascending phase of the next cycle, the fourth for phase of maximum, and the last one for the descending phase of the new cycle. With an antisymmetric Babcock–Leighton (BL) source, the last image will have the same field configuration as the first, but with all field lines reversed. Within Figures 8 and 9 we have also placed an observed coronal image that resembles most closely the dynamo model image for a similar cycle phase.

Figure 8 shows our reference case for these simulations. Here the antisymmetry is preserved through a sunspot cycle. We see the evolution of the corona from descending phase (panel a) to minimum (panel b), at which the dipole field lines dominate, through the ascending phase (panel c) to maximum (panel d), during which higher multipoles develop. Since antisymmetry about the Equator is maintained, the first multipole is what we have called the hexapole (as opposed to the quadrupole, which has the opposite symmetry). In Figure 8e we show the corona during the minimum of 1996, a typical minimum corona, to compare with Figure 8b directly above it. Finally, we complete the sunspot cycle in Figure 8f, which shows the descending phase of the new cycle, which is very similar to Figure 8a but with all the poloidal-field lines, and therefore color shading, reversed.

Figure 9 tells quite a different story. Here, solar activity ends in the North about two years ahead of the South, represented in this case by truncating the surface poloidal source at low latitudes in the North. Figure 9a shows the corona at the time when the asymmetry is introduced. In 3.2 years, we see that the corona at sunspot minimum (Figure 9b) no longer looks like the typical minimum corona with a dipole configuration. The North looks somewhat like a dipole, but the South retains higher-order multipoles, at least quadrupole, hexapole, and even octupole components, and the whole configuration is quite asymmetric about the Equator. This asymmetry extends into the ascending phase of the next cycle (Figure 9c), which shows that the polar field has reversed in the North, but not yet in the South, indicating that at this time the quadrupole component is important.

By the time of the next maximum, the South polar field has reversed, and the whole configuration is again nearly antisymmetric about the Equator. But because of the low and mid-latitude fields that generate coronal structures with smaller latitudinal scale, this maximum field is not dipolar. There is also a substantial hexapolar component. In Figure 9e, which shows the next descending phase, we can see small differences compared to Figure 9a, carried over from the difference in surface poloidal source late in the previous cycle. These differences will again enlarge to repeat the significant coronal asymmetry as the next minimum is approached. Figure 9f shows the corona in 2008, the atypical minimum just passed. We see that it compares best with both the minimum and the ascending phase, since none of them is really dipolar.

## Comparisons to Earlier Works

Wang and Sheeley (2003) carried out a detailed study of the evolution of coronal structures through a solar cycle, estimated by application of a surface flux-transport model containing latitudinal diffusion and transport by meridional circulation acting on ‘rings’ or axisymmetric zones of bipolar magnetic regions characteristic of Cycle 21. As with our dynamo model, their surface model was coupled to a coronal source-surface model, but also has no longitude dependence in it. The starting point for their calculations was solar minimum with a specified purely dipole field. For most of their simulations antisymmetry about the Equator was maintained but they also simulated six years of Cycle 21 without this assumption, for the years 1977 – 1983.

Aside from the different models used, their simulations were focused on a different goal, namely to study in detail how the corona changes as the polar fields reverse near solar maximum, while our focus has been on explaining the atypical minimum coronal structures seen near the end of Cycle 23. Their study showed the evolution of the occurrence of X-type neutral points in the corona as the cycle progresses through the polar field reversal, something we have not attempted. On the other hand, they did not attempt to compare the details of their coronal simulations with white-light coronal images for the period, or compute the dominant Legendre polynomial modes.

The coronal structures found from their model and ours are qualitatively similar, even though the time periods being simulated do not overlap. They are able to capture the North–South asymmetry in coronal structures characteristic of Cycle 21, but that is a much extreme case than for the latter part of Cycle 23, which we have focused on. Nevertheless it seems clear that the same atypical coronal structures we have simulated could also be captured using their surface flux-transport model.

More recently, DeRosa, Brun, and Hoeksema (2012) and Petrie (2013) have carried out modal analyses of surface magnetic fields for the period 1977 – 2012 to determine the evolution of the relative strengths of various spherical harmonic modes through a solar cycle. These analyses included modes with longitude dependence, but since our focus has been exclusively on axisymmetric structures, we will compare our results to those in DeRosa, Brun, and Hoeksema (2012) and Petrie (2013) mainly for the axisymmetric case. Certain aspects of the evolution of the amplitudes of these modes and their coupling through a solar cycle were studied by DeRosa, Brun, and Hoeksema (2012) and Petrie (2013) using low-order 1D nonlinear dynamo models as well as a flux-transport dynamo model.

The closest point of comparison between our results and those in DeRosa, Brun, and Hoeksema (2012) is between the axisymmetric spherical harmonics they find and the Legendre polynomial amplitudes we infer from coronal white-light images. We see that they both show that, in qualitative terms at least, the axial dipole contains the largest fraction of magnetic energy in the corona near solar minimum, but in the more active phases of the cycle the energy in the quadrupole and higher multipoles can be several times larger than in the dipole. We infer that if DeRosa, Brun, and Hoeksema (2012) had computed coronal structures from their surface field modes, they would have found coronal structures similar to ours for various cycle phases. We can see this correspondence directly in the PFSS models calculated from related spherical harmonic magnetic data in Petrie (2013). The DeRosa, Brun, and Hoeksema (2012) mean field BL type dynamo calculations confirm the predominance of the dipole component near solar minimum, but higher multipole components near maximum, in the same way ours did. They also showed that only a small amount of asymmetry in meridional circulation between the North and South hemispheres is enough to give significant amplitude to the quadrupole fields through most of a cycle.

## Discussion and Conclusions

Our primary result is that low-order axisymmetric Legendre polynomials can be used to represent white-light coronal structures that are similar to the output of a flux-transport dynamo coupled with a potential-field source-surface model, as these structures evolve over a solar cycle, and differ for minima of different cycles. The main limitation of this approach is the absence of longitude dependence in the polynomials that could capture the presence of different coronal structures at the same time in the East and West limbs of the Sun. In detail, we have qualitatively fitted low-order axisymmetric Legendre polynomials for poloidal-field potentials [$A$] to white-light global coronal structures for various phases of the past three solar cycles, and shown that only a few such polynomials are needed to get reasonable correspondence between the two limbs. We have confirmed that for the previous two cycle minima the dipole polynomial is enough, but a higher-order term is needed for the 2008 minimum. We carried out simulations of the global coronal structure using a calibrated solar flux-transport dynamo model coupled to a potential-field source-surface model for the corona to find a possible physical cause for the absence of a dipole-like coronal minimum in 2008. We showed that when the surface poloidal-field source is made to decline to near zero two years earlier in the North than in the South, as was observed, the coronal structure passes through a minimum phase without ever being a pure dipole.

This result suggests that the origin of the non-dipole configuration in the latest minimum is due to the combination of phase and amplitude differences in solar activity between North and South in Cycle 23. The cause of such phase and amplitude differences between hemispheres in terms of differences in dynamo ingredients is currently an active area of investigation. One probable possibility is that there are differences between North and South in meridional circulation amplitude, which can lead to both cycle amplitude differences and particularly cycle phase differences between North and South (see, *e.g.* Belucz and Dikpati 2013). Both of these differences break the antisymmetry of poloidal fields about the Equator, necessarily implying a significant amplitude in the quadrupolar component.

These results suggest that if the North continues to lead the South in Cycle 24 we might expect that at the minimum between Cycles 24 and 25 the corona would again be rather non-dipolar. It would also be worthwhile to expand the study by looking at coronal images and their fits to low-order polynomials for as many past cycles as possible, to see whether non-dipolar configurations at solar minimum exist for earlier minima and, if so, whether these are also associated with phase differences between the North and South hemispheres in surface poloidal flux. Another possibility is the asymmetry in the inflow cells on the average meridional circulation, caused by the amount of emerging flux differed in the North and South hemispheres (see, *e.g.*, Shetye, Tripathi, and Dikpati 2015).

As indicated in the introduction, a limitation of the results presented here is confining the analysis to axisymmetric features. Ultimately it will be necessary to consider longitude-dependent coronal structures to compare with the output from a flux-transport dynamo model that has been generalized to include global departures from axisymmetry to include low longitudinal wavenumbers, say $m=1,2$ at least, but still coupled with a source-surface coronal field model. Both DeRosa, Brun, and Hoeksema (2012) and Petrie (2013) put strong emphasis on the role of modes with longitude dependence in determining the wandering of the Sun’s magnetic axis and the existence of sector structures. To estimate the associated Legendre polynomials associated with such longitude-dependent coronal structures will require white-light coronal observations much more closely spaced in time, which do exist in the archives of MLSO for the past several solar cycles.
